# Relationship between serum indirect bilirubin levels and skeletal muscle mass in older male and female patients with type 2 diabetes

**DOI:** 10.1371/journal.pone.0276976

**Published:** 2022-11-02

**Authors:** Yukihiro Inoguchi, Toyoshi Inoguchi, Tomoaki Eto, Mitsunori Masakado, Satoru Suehiro, Teruaki Yamauchi, Fumio Umeda

**Affiliations:** 1 Yukuhashi Central Hospital, Yukuhashi, Japan; 2 Division of Endocrinology and Metabolism, Department of Internal Medicine, Kurume University School of Medicine, Kurume, Japan; 3 Fukuoka City Health Promotion Support Center, Fukuoka City Medical Association, Fukuoka, Japan; Maastricht University Faculty of Health, Medicine and Life Sciences: Maastricht Universitair Medisch Centrum+, NETHERLANDS

## Abstract

**Objective:**

We previously showed that low serum bilirubin levels are associated with disability in quality of daily living in older patients with diabetes. However, the underlying mechanism is not fully understood. The aim of this study is to assess the relationship between serum bilirubin levels and skeletal muscle mass in older patients with type2 diabetes.

**Methods:**

A total of 272 older patients with type2 diabetes (152 male and 120 female) aged 60 years and over were continuously recruited from April 2020 to July 2020. Body composition was evaluated by bioelectrical impedance analysis. The skeletal muscle mass index (SMI) was calculated as appendicular muscle mass divided by height squared (m^2^).

**Results:**

The SMI was markedly lower in old-old patients (aged 75 years and over) than in young-old patients (aged 60–74 years) in both male and female (7.1 ± 0.8 kg/m^2^ vs 7.6 ± 0.9 kg/m^2^, P<0.001; 5.5 ± 0.9 kg/m^2^ vs 6.3 ± 0.8 kg/m^2^, P<0.001, respectively). Multivariate regression analysis showed that the SMI was associated with body mass index (BMI) (p<0.001) and age (p = 0.048) in male young-old patients, while it was associated with BMI (p<0.001), age (p = 0.008), and serum indirect bilirubin levels (p = 0.038) in male old-old patients. In female, the SMI was associated with BMI (p<0.001) and age (p = 0.042) in young-old patients and associated with BMI alone (p<0.001) in old-old patients.

**Conclusion:**

Serum indirect bilirubin levels may be associated with the decreased skeletal muscle mass in male older patients (aged 75 years and over) with type 2 diabetes.

## Introduction

In ageing societies such as Japan and many Western countries, the prevalence of frailty and its adverse outcomes increases. Disability in activity of daily living (ADL) is an adverse outcome of frailty and it places a high burden on frail people, care professionals, and health care system. Older patients with diabetes are at the risk of frailty and frail individuals with diabetes have a higher mortality than non-frail individuals with diabetes [1, 2]. Therefore, it is important to know which factors predict frailty and ADL disability in older individuals with diabetes. In recent years, oxidative stress has received attention as one of the important causative factors of frailty, and oxidative stress and proinflammatory biomarkers were reported to be increased in physically frail and prefrail subjects [3–5]. Bilirubin is a strong endogenous antioxidant [6]. Accumulating evidence has shown the relationship between serum bilirubin levels and oxidative stress-related diseases such as diabetes, diabetic nephropathy, and cardiovascular diseases [7–12]. In addition, in the previous report, we showed that serum levels of bilirubin is a strong predictive biomarker for ADL disability in older patients with diabetes (aged 70 years and over) [13]. However, the mechanisms underlying the link between serum bilirubin levels and ADL disability is not fully elucidated.

Loss of skeletal muscle mass or sarcopenia is one of the main features of frailty and ADL disability [14, 15]. Many studies have shown that diabetes is a risk factor for sarcopenia [16–19]. Since oxidative stress is thought to be involved in age-related skeletal muscle abnormalities through various pathways [20, 21], it may also play a role in diabetes-induced skeletal muscle abnormalities [16]. Therefore, the objective of this study is to evaluate whether serum bilirubin levels are associated with skeletal muscle mass in older patients with diabetes.

## Material and methods

### Participants

A total of 272 older patients (152 male and 120 female) aged 60 years and older with type 2 diabetes under medical care at Yukuhashi Central Hospital (Japan) were continuously recruited from April 2020 to July 2020. Patients who suffered from liver cirrhosis, other hepatobiliary diseases with abnormal liver enzyme levels (alanine aminotransferase or alkaline phosphatase levels > 2-fold of the upper limit of the normal range), or hemolytic anemia were excluded to evaluate the true relationship of serum bilirubin levels with the skeletal muscle mass. In this study, the participants were divided to two groups, according to their ages, namely, young-old (60–74 years old) and old-old (75 years old and over) groups. All procedures were performed in accordance with the relevant guidelines and regulations. Informed consent was obtained in the form of opt-out. Those who rejected were excluded. The study was approved by the ethics committee of Yukuhashi Central Hospital (No. 20220113034).

### Clinical variables and definition

Body composition was evaluated by bioelectrical impedance analysis (BIA). All participants were subjected to a BIA analyzer (Inbody S10, InBody Japan, Tokyo, Japan) with a segmental multifrequency approach (1 kHz, 5 kHz, 50 kHz, 250 kHz, 500 kHz, and 1 MHz). Inbody analyzer was reported to have a good correlation with dual-energy X-ray absorptiometry [22]. Appendicular muscle mass, fat mass, and body fat mass percent was obtained using this analyzer. The skeletal muscle mass index (SMI) was calculated as appendicular muscle mass divided by the square of body height in meters. Peripheral blood samples were collected after overnight fasting. Both of total and direct serum bilirubin level was measured by the vanadate oxidation method [23]. Indirect bilirubin level was measured as the difference between total and direct bilirubin level. Hemoglobin A1c (HbA1c) value was determined using a standard high-performance liquid chromatography method and presented as the National Glycohemoglobin Standardization Program value. The estimated glomerular filtration rate (eGFR) was calculated with an equation from the Japanese Society of Nephrology [24]. Hypertension was defined as systolic blood pressure >140 mmHg or diastolic blood pressure >90 mmHg, or the current use of any antihypertensive medication. Hyperlipidemia was defined as serum concentration of LDL cholesterol ≥120 mg/dL and triglyceride ≥150 mg/dL in accordance with the Japan Atherosclerosis Society criteria, or the current use of lipid-lowering agents. Albuminuria was defined as a urinary albumin/creatinine ratio of ≥30 mg/g creatinine. Chronic renal failure was defined as an estimated glomerular filtration rate (eGFR) < 30 mL/min/1.73 m^2^. Coronary artery diseases were defined as a history of acute myocardial infarction or angina pectoris confirmed by clinically significant obstruction on coronary angiography or revascularization with angioplasty or coronary artery bypass. Low SMI was defined as SMI < 7.0 kg/m^2^ in men and SMI <5.7 kg/m^2^ in women in accordance with the criteria of sarcopenia in the Asian Working Group for Sarcopenia (AWGS).

### Statistical analysis

Continuous variables were presented as mean ± standard deviation (SD) and categorical variables were presented as numbers and percentages. Significance of differences was determined by the unpaired t test or the Mann-Whitney U test for the continuous variables and the chi-squared test for categorical variables. Univariate analysis was performed with Spearman rank correlation. Multiple regression analysis was used to determine the relationship between the SMI and serum bilirubin levels and other variables which were significantly correlated with the SMI in univariate analysis. Statistical analysis was performed using BellCurve for Excel version 3.21 (Tokyo, Japan). A two-sided P value < 0.05 was considered to be significant.

## Results

The characteristics of 272 older patients (152 male and 120 female) with diabetes enrolled in our study are shown in Table 1. The mean age was 72.9 ± 7.6 years in male and 74.5 ± 8.6 years in female. The mean body mass index (BMI) was 24.0 ± 3.2 kg/m^2^ in male and 24.3 ± 4.9 kg/m^2^ in female. In general, skeletal muscle mass is lower in females versus males, and cutoffs for low/pathological muscle mass are different for males and females. Therefore, we performed analyses separately in male and female. In addition, people with ADL disability and requiring long-term care significantly increases after the age of 75 years in Japan (www.jili.or.jp/lifeplan/lifesecurity/1118.html). In this study, we therefore performed analyses separately in young-old (aged 60-74years) and old-old (aged 75years and over). The SMI was lower in old-old patients than in young-old patients in male (7.1 ± 0.8 kg/m^2^ vs 7.6 ± 0.9 kg/m^2^, P<0.001) and in female (5.5 ± 0.9 kg/m^2^ vs 6.3 ± 0.8 kg/m^2^, P<0.001). In addition, the percentage of low SMI which was in accordance with the criteria of sarcopenia (SMI < 7.0 kg/m^2^ in men and SMI <5.7 kg/m^2^ in women) was higher in old-old patients than in young-old patients in male (44% vs 27%, P = 0.030) and in female (65% vs 20%, P<0.001). The indirect serum bilirubin levels were lower in old-old patients than in young-old patients in both male and female (0.4 mg/dL [0.3–0.4] vs 0.4 mg/dL [0.3–0.6], P<0.001; 0.3 mg/dL [0.3–0.5] vs 0.4 mg/dL [0.3–0.5], P = 0.030, respectively).

**Table 1:** Clinical characteristics of patients.

	Male				Female			
Variables	All	Young-old (aged 60–74)	Old-old (aged ≥75)	p-value	All	Young-old (aged 60–74)	Old-old (aged ≥75)	p-value
	n = 152	n = 90	n = 62		n = 120	n = 66	n = 54	
Age (years)	72.9±7.6	67.6±4.1	80.4±4.6	<0.001^a^	74.5±8.6	67.8±4.0	82.6±5.1	<0.001^a^
Height (cm)	164.1±6.4	165.2±6.8	162.5±5.4	0.011^a^	150.3±5.7	152.8±5.0	147.2±5.1	<0.001^a^
Weight (kg)	64.7±10.4	66.1±11.4	62.8±8.4	0.058^a^	54.8±11.4	57.5±12.6	51.5±8.9	0.004^a^
BMI (kg/m2)	24.0±3.2	24.1±3.4	23.8±2.8	0.483^a^	24.3±4.9	24.7±5.4	23.8±4.1	0.331^a^
SLM (kg)	43.8±6.1	45.6±6.3	41.2±4.8	<0.001^a^	32.4±4.4	34.4±4.0	30.0±3.6	<0.001^a^
SMM (kg)	25.1±3.9	26.4±4.0	23.3±3.0	<0.001^a^	17.9±2.8	19.3±2.5	16.3±2.2	<0.001^a^
FFM (kg)	46.3±6.4	48.2±6.6	43.5±5.1	<0.001^a^	34.4±4.5	36.4±4.1	31.9±3.7	<0.001^a^
SMI (kg/m2)	7.4±0.9	7.6±0.9	7.1±0.8	<0.001^a^	5.9±0.9	6.3±0.8	5.5±0.9	<0.001^a^
Low SMI n (%)	51 (34)	24 (27)	27 (44)	0.030^c^	48 (40)	13 (20)	35 (65)	<0.001^c^
BFP (%)	27.9±7.3	26.3±7.3	30.2±6.8	0.001^a^	36.0±8.5	35.2±8.7	37.0±8.4	0.253^a^
Fat (kg)	18.4±6.5	17.9±6.9	19.3±6.0	0.200^a^	20.5±8.7	21.2±9.6	19.8±7.4	0.381^a^
HbA1c (%)	7.0 (6.5–7.6)	7.0 (6.5–7.5)	7.0 (6.4–7.6)	0.731^b^	7.0 (6.6–7.7)	7.0 (6.6–7.6)	7.2 (6.7–8.0)	0.124^b^
Total protein (g/dL)	7.2 (6.9–7.5)	7.2 (6.9–7.5)	7.1 (6.8–7.5)	0.482^b^	7.2 (6.8–7.5)	7.3 (6.8–7.6)	7.2 (6.8–7.4)	0.175^b^
Albumin (g/dL)	4.3 (4.1–4.5)	4.4(4.2–4.6)	4.2 (4.1–4.4)	<0.001^b^	4.4 (4.1–4.5)	4.4 (4.3–4.6)	4.2 (4.0–4.4)	<0.001^b^
T-bilirubin (mg/dL)	0.6 (0.5–0.8)	0.6 (0.5–0.8)	0.6 (0.5–0.7)	0.074^b^	0.5 (0.4–0.7)	0.5 (0.4–0.7)	0.5 (0.4–0.6)	0.245^b^
ID-bilirubin (mg/dL)	0.4 (0.3–0.5)	0.4 (0.3–0.6)	0.4 (0.3–0.4)	<0.001^b^	0.4 (0.3–0.5)	0.4 (0.3–0.5)	0.3 (0.3–0.5)	0.030^b^
Hypertension n (%)	113 (74)	61 (68)	52 (84)	0.026^c^	84 (70)	42 (64)	42 (78)	0.093^c^
Hyperlipidemia n (%)	113 (74)	67 (74)	46 (74)	0.972^c^	100 (83)	53 (80)	47(87)	0.325^c^
CAD n (%)	28 (18)	10 (11)	18 (29)	0.005^c^	16 (13)	4 (6)	12 (22)	0.010^c^
CVD n (%)	13 (9)	5 (6)	8 (13)	0.111^c^	10 (8)	6 (9)	4 (7)	0.740^c^
Albuminuria n (%)	69/147 (47)	38/86 (44)	31/61 (51)	0.427^c^	54/111 (49)	21/61 (34)	33/50 (66)	<0.001^c^
Renal failure n (%)	11 (7)	4 (4)	7 (11)	0.109^c^	9 (8)	5 (8)	4 (7)	0.972^c^
DPP4i use n (%)	101 (66)	63 (70)	38 (61)	0.264^c^	66 (55)	37 (56)	29 (54)	0.796^c^
SGLT2i use n (%)	29 (19)	23 (26)	6 (10)	0.014^c^	17 (14)	13 (20)	4 (7)	0.055^c^
SU use n (%)	36 (24)	18 (20)	18 (29)	0.198^c^	28 (23)	10 (15)	18 (33)	0.019^c^
Metformin use n (%)	59 (39)	41 (46)	18 (29)	0.040^c^	45 (38)	27 (41)	18 (33)	0.394^c^
GLP-1RA use n (%)	19 (13)	10 (11)	9 (15)	0.533^c^	21 (18)	9 (14)	12 (22)	0.218^c^
Insulin use n (%)	30 (20)	14 (16)	16 (26)	0.119^c^	22 (18)	7 (11)	15 (28)	0.016^c^

^a^Calculated using the t test.

^b^Calculated using the Mann–Whitney U test.

^c^Calculated using the chi-squared test.

P value, comparison was performed between young-old patients and old-old patients.

Missing number of patients: Albumin: all n = 8, young-old n = 1, old-old n = 7 in male; all n = 10, young-old n = 3, old-old n = 7 in female. ID-bilirubin: all n = 8, young-old n = 1, old-old n = 7 in male; all n = 12, young-old n = 5, old-old n = 7 in female. Albuminuria: all n = 5, young-old n = 4, old-old n = 1 in male; all n = 9, young-old n = 5, old-old n = 4.

Abbreviation: BMI, body mass index; SMI, skeletal muscle mass index; SLM, soft lean mass; SMM, skeletal muscle mass; FFM, fat free mass; BFP, body fat percent; HbA1c, hemoglobin A1c; T-bilirubin, total bilirubin; ID-bilirubin, indirect bilirubin; CAD, cardiovascular disease; CVD, cerebrovascular disease; DPP4i, dipeptidyl peptidase-4 inhibitor; SGLT2i, sodium glucose co transporter-2 inhibitor; SU, sulfonylurea; GLP1-RA, glucagon-like peptide-1 receptor agonists.

In univariate analysis, the SMI was positively correlated with BMI (p<0.001) and negatively correlated with age (p = 0.031) in male young-old patients, while it was positively correlated with BMI (p<0.001), serum total bilirubin levels (P = 0.012) and indirect bilirubin levels (p = 0.004), and negatively correlated with age (p = 0.012), HbA1c (p<0.001), a history of coronary artery disease (p = 0.011), and use of sulfonylurea drugs (p = 0.029) in male old-old patients (Table 2). In female, the SMI was positively correlated with BMI (p<0.001), body fat percentage (p<0.001), hypertension (P = 0.004) and hyperlipidemia (P = 0.014), and negatively correlated with serum total bilirubin (P = 0.045) in young-old patients (Table 3). It was positively correlated with BMI alone (P<0.001) in old-old patients (Table 3).

**Table 2:** Univariate analysis of factors associated with skeletal muscle mass index (SMI) in male.

	All		Young-old (aged 60–74)		Old-old (aged 75≤)	
	n = 152		n = 90		n = 62	
Variables	r	p-value	r	p-value	r	p-value
Age	**-0.363**	**<0.001**	**-0.228**	**0.031**	**-0.317**	**0.012**
BMI	**0.677**	**<0.001**	**0.752**	**<0.001**	**0.589**	**<0.001**
BFP	0.033	0.685	0.203	0.055	-0.015	0.909
HbA1c	**-0.180**	**0.026**	-0.047	0.659	**-0.453**	**<0.001**
Total protein	-0.073	0.372	-0.171	0.107	0.057	0.662
Albumin	0.092	0.274	-0.094	0.383	0.224	0.100
T-bilirubin	0.088	0.280	-0.096	0.371	**0.318**	**0.012**
ID-bilirubin	0.133	0.111	-0.093	0.389	**0.383**	**0.004**
Hypertension	0.101	0.215	0.175	0.099	0.147	0.254
Hyperlipidemia	-0.105	0.200	-0.108	0.309	-0.087	0.504
CAD	**-0.259**	**0.001**	-0.097	0.365	**-0.322**	**0.011**
CVD	-0.062	0.449	-0.029	0.787	-0.008	0.950
Albuminuria	0.003	0.975	-0.057	0.605	0.153	0.240
Renal failure	0.079	0.333	-0.010	0.923	0.246	0.054
DDP4i	-0.006	0.940	-0.060	0.573	0.020	0.875
SGLT2i	0.140	0.085	0.148	0.165	-0.031	0.814
SU	-0.144	0.076	0.009	0.936	**-0.278**	**0.029**
Metformin	0.108	0.187	0.130	0.222	-0.036	0.783
GLP-1RA	-0.106	0.194	-0.038	0.721	-0.180	0.161
Insulin	-0.103	0.206	-0.120	0.259	-0.027	0.836

Spearman rank correlation test was performed to evaluate the correlation between skeletal muscle mass index

(SMI) and various variables.

Number of patients with missing data: Albumin, all n = 8, young-old n = 1, old-old n = 7; ID-Bil, all n = 8, young-old n = 1, old-old n = 7; Albuminuria, all n = 5, young-old n = 4, old-old n = 1.

Abbreviation: BMI, body mass index; SMI, skeletal muscle mass index; BFP, body fat percent; HbA1c, hemoglobin A1c; T-bilirubin, total bilirubin; ID-bilirubin, indirect bilirubin; CAD, cardiovascular disease; CVD, cerebrovascular disease; DPP4i, dipeptidyl peptidase-4 inhibitor; SGLT2i, sodium glucose co transporter-2 inhibitor; SU, sulfonylurea; GLP1-RA, glucagon-like peptide-1 receptor agonists.

**Table 3:** Univariate analysis of factors associated with skeletal muscle mass index (SMI) in female.

	All		Young-old (aged 60–74)		Old-old (aged 75≤)	
	n = 120		n = 66		n = 54	
Variables	r	p-value	r	p-value	r	p-value
Age	**-0.433**	**<0.001**	-0.171	0.170	-0.144	0.299
BMI	**0.614**	**<0.001**	**0.742**	**<0.001**	**0.581**	**<0.001**
BFP	**0.249**	**0.006**	**0.469**	**<0.001**	0.192	0.164
HbA1c	0.004	0.970	0.026	0.839	0.198	0.150
Total protein	0.111	0.226	0.138	0.269	0.018	0.899
Albumin	0.186	0.052	0.103	0.424	-0.080	0.593
T-bilirubin	-0.041	0.660	**-0.248**	**0.045**	0.064	0.646
ID-bilirubin	0.005	0.955	-0.213	0.100	0.052	0.728
Hypertension	0.122	0.185	**0.347**	**0.004**	0.037	0.790
Hyperlipidemia	0.108	0.238	**0.301**	**0.014**	-0.012	0.929
CAD	-0.021	0.818	-0.013	0.915	0.112	0.422
CVD	0.093	0.312	-0.017	0.895	0.186	0.178
Albuminuria	-0.147	0.124	-0.110	0.400	0.142	0.326
Renal failure	0.046	0.617	0.086	0.494	0.032	0.820
DDP4i	-0.034	0.714	-0.193	0.120	0.130	0.349
SGLT2i	**0.185**	**0.044**	0.213	0.086	0.018	0.896
SU	-0.141	0.124	-0.169	0.176	-0.013	0.928
Metformin	0.027	0.769	-0.083	0.506	0.066	0.638
GLP-1RA	-0.002	0.986	0.180	0.149	-0.089	0.524
Insulin	-0.157	0.086	-0.136	0.278	0.036	0.797

Spearman rank correlation test was performed to evaluate the correlation between skeletal muscle mass index

(SMI) and various variables.

Number of patients with missing data: Albumin, all n = 10, young-old n = 3, old-old n = 7; ID-bilirubin, all n = 12, young-old n = 5, old-old n = 7; Albuminuria, all n = 9, young-old n = 5, old-old n = 4.

Abbreviation: BMI, body mass index; SMI, skeletal muscle mass index; BFP, body fat percent; HbA1c, hemoglobin A1c; T-bilirubin, total bilirubin; ID-bilirubin, indirect bilirubin; CAD, cardiovascular disease; CVD, cerebrovascular disease; DPP4i, dipeptidyl peptidase-4 inhibitor; SGLT2i, sodium glucose co transporter-2 inhibitor; SU, sulfonylurea; GLP1-RA, glucagon-like peptide-1 receptor agonists.

Next, multivariate linear regression analysis was performed to evaluate the relationship between the SMI and serum bilirubin levels. We selected serum indirect bilirubin levels, age, BMI, HbA1c and other variables which were significantly correlated with the SMI in univariate analysis as explanatory variables. However, in young-old female, serum indirect bilirubin levels and body fat percent were excluded from explanatory variables because they were strongly correlated with total bilirubin levels and BMI, respectively. As results, the SMI was correlated with BMI (p<0.001) and age (p = 0.048) in male young-old patients, while it was correlated with BMI (p<0.001), age (p = 0.008) and serum indirect bilirubin levels (p = 0.038) in male old-old patients (Table 4). In female, the SMI was correlated with BMI (p<0.001) and age (p = 0.042) in young-old patients, and it was correlated with BMI alone (p<0.001) in old-old patients (Table 4).

**Table 4:** Determinants for skeletal muscle mass index (SMI) evaluated by multivariate linear regression analysis.

**Male**			
**Young-old**	**Variables**	**β**	**p-value**
	Age	**-0.142**	**0.048**
	BMI	**0.735**	**<0.001**
	HbA1c	-0.077	0.293
	ID-bilirubin	-0.027	0.711
**Old-old**	**Variables**	**β**	**p-value**
	Age	**-0.274**	**0.008**
	BMI	**0.482**	**<0.001**
	HbA1c	-0.185	0.090
	ID-bilirubin	**0.203**	**0.038**
	CAD	-0.164	0.111
	SU	-0.088	0.382
**Female**			
**Young-old**	**Variables**	**β**	**p-value**
	Age	**-0.151**	**0.042**
	BMI	**0.772**	**<0.001**
	HbA1c	-0.053	0.450
	T-bilirubin	-0.072	0.338
	Hypertension	0.060	0.428
	Hyperlipidemia	0.037	0.617
**Old-old**	**Variables**	**β**	**p-value**
	Age	-0.167	0.213
	BMI	**0.493**	**<0.001**
	HbA1c	0.026	0.848
	ID-bilirubin	0.055	0.679

Abbreviation: BMI, body mass index; SMI, skeletal muscle mass index; HbA1c,

hemoglobin A1c; ID-bilirubin, indirect bilirubin; T-bilirubin, total bilirubin

β shows standardized coefficient

## Discussion

Elderly subjects with diabetes are at increased risk of frailty and ADL disability [1, 2]. Therefore, it is clinically useful to identify the predictable and potentially treatable biomarker for these conditions. In this study, we found that indirect bilirubin levels were significantly correlated with SMI in male old-old diabetic patients (aged 75 years old and over), but not in young-old males, or in females.

We previously reported that low serum bilirubin level is a strong predictive biomarker for ADL disability in older patients (aged 70 years and over) with diabetes. In the present study, we examined the association between serum bilirubin levels and skeletal muscle mass, which is one of the main features for ADL disability, in older patients with diabetes. The present study showed that the SMI was markedly lower in old-old patients (aged 75 year and over) with diabetes than young-old patients with diabetes (aged 60–74 years) in both male and female. Although age and BMI were the strongest determinants for the SMI in older patients in both male and female, serum indirect bilirubin levels were positively correlated with the SMI in male old-old patients. These results suggest that serum indirect bilirubin levels may be associated with the decreased skeletal muscle mass in old-old patients with type 2 diabetes.

Type 2 diabetes is associated with increased risk of sarcopenia [16–19]. It has been postulated that metabolic abnormalities in type 2 diabetes may be involved in sarcopenia, including insulin resistance, hyperglycemia, inflammation and overproduction of reactive oxygen species (ROS). Among them, overproduction of ROS are thought to be common causative factors for the loss of muscle quantity and quality [21, 25]. As for the muscle quantity, overproduction of ROS may activate the ubiquitin-proteasome system and muscle proteases (caspases, calpains), leading to protein degradation. In muscle satellite cells and myoblasts, ROS are reported to increase nuclear factor-kappa B (NF-κβ) activity [26]. This may cause a reduction of expression of MyoD which is necessary for proliferation and induction of differentiation [27]. In addition, ROS may induce apoptosis of progenitor and mature skeletal muscle cells [28].

Bilirubin is a strong endogenous antioxidant. Many epidemiological and animal studies have shown that serum bilirubin levels are inversely associated with the onset and development of oxidative stress-related diseases including diabetes, diabetic vascular complications, chronic kidney disease (CKD), and cardiovascular diseases [7–12]. Therefore, it is most likely that the negative relationship between serum indirect bilirubin levels and the skeletal muscle mass in male old-old patients with type 2 diabetes is due to the decreased antioxidative activity and subsequent increased oxidative stress. Such relationship with serum indirect bilirubin levels was more evident than that with total bilirubin levels. One possible explanation for this is that direct bilirubin in serum is rapidly secreted into bile and most of total bilirubin in serum is indirect bilirubin under normal conditions. Another one is that indirect bilirubin, but not direct bilirubin, can serve as an antioxidant inside the cells because indirect bilirubin is lipophilic and can cross the plasma membrane, although both forms of bilirubin have antioxidant activities.

We also found that SMI was negatively correlated with CAD in old-old male patients in univariate analysis. This was consistent with the finding that sarcopenia is associated with CAD [29]. However, this correlation could not be seen in multiple regression analysis, and we can’t distinguish whether this correlation is direct or indirect. It is possible that both decreased SMI and CAD may be mediated by decreased serum bilirubin levels.

In the present study, such relationship was limited to male, but not in female. The reason for the sex difference was not clear. However, it is possible that serum bilirubin levels may negatively affect muscle quality rather than quantity in female, because age-related muscle abnormalities are reported to occur predominantly in muscle quantity in male, while they occur predominantly in muscle quality in female [30]. In fact, there was no sex difference in the relationship between serum bilirubin levels and ADL disability in our previous report. Another possibility may be explained by sex hormone. Testosterone declines with age, and it plays roles in regulating muscle mass and fat mass [31]. Of interest, recent report showed that low serum bilirubin levels are associated with testosterone deficiency in older male [32]. The detailed underlying mechanism remained to elucidated, although it was speculated that low levels of testosterone and bilirubin may be closely interrelated via oxidative stress and chronic low-grade inflammation [32]. Taken together, it is possible that low serum bilirubin levels might be associated with decreased skeletal muscle mass via the testosterone deficiency in old-old male patients. The detailed mechanisms underlying the sex difference should be evaluated in future studies.

This study has some limitations. First, this study was carried out at single institute and the sample size was small. Therefore, selection bias could not be completely excluded. Second, the cross-sectional design is not suited to study the causal effect. Third, we could not obtain information on oxidative stress markers, nutrition and exercise which are important risk factors for sarcopenia. The lack of these data raised the possibility of unmeasured confounders and weakened the results of this study. Fourth, we measured body composition and SMI by only BIA and did not measure them by a reference method such as dual-energy X-ray absorptiometry.

In conclusion, serum indirect bilirubin levels were positively correlated with the SMI in old-old patients with type 2 diabetes, suggesting that serum indirect bilirubin levels may be associated with the decreased skeletal muscle mass in male old-old patients with type 2 diabetes. The causal relationship and its clinical utility should be evaluated in future prospective studies.

## Supporting information

S1 File (PDF)
