# Supplementary material for: Relationship between serum indirect bilirubin levels and skeletal muscle mass in older male and female patients with type 2 diabetes
Source: PLoS One. 2022 Nov 2;17(11):e0276976. doi: 10.1371/journal.pone.0276976 (PMC9629548; doi:10.1371/journal.pone.0276976)
Supplement: S1 File — (PDF) [file pone.0276976.s001.pdf]

Patient No.	Age	Sex（male 0）	HbA1c	T-bilirubin	ID-bilirubin	Total protein
1	60	0	9.5	0.7	0.5	7.1
2	60	0	5.9	1.1	0.8	6.9
3	60	0	6.9	0.6	0.4	7.4
4	60	0	5.4	0.6	0.4	7.1
5	60	1	7.3	1.2	0.9	6.7
6	61	0	6.9	0.7	0.4	7.3
7	61	0	7.3	0.3	0.2	7.2
8	61	1	7.5	0.7	0.5	7.6
9	61	1	6.1	0.6	0.5	6.8
10	62	0	6	1.8	1.2	6.4
11	62	0	7.6	0.5	0.3	6.8
12	62	0	6.5	1.3	0.9	7.3
13	62	0	7.6	0.4	0.3	5.8
14	62	0	6.5	1.6	1.2	7.7
15	62	0	6.7	0.8	0.6	7.3
16	62	0	6.6	0.8	0.6	7.4
17	62	1	6.2	0.8	0.5	8.2
18	62	1	5.7	0.6	0.4	7.3
19	62	1	6.6	0.9	0.7	7.6
20	62	1	8	0.5	0.4	8.3
21	62	1	6.6	0.7	0.5	7.6
22	63	0	6.1	0.7	0.5	7.6
23	63	0	6.4	1.3	0.9	7.4
24	63	0	8.3	0.7	0.4	7
25	63	0	7.8	1.1	0.9	7.6
26	63	0	6.2	0.7	0.5	6.9
27	63	0	7.1	0.4	0.2	7.4
28	63	1	7	0.3	0.2	7.5
29	63	1	7.1	0.4	0.3	7.5
30	63	1	7.4	0.7	0.5	6.8
31	63	1	8.2	0.4	0.3	7.2
32	64	0	7.1	0.6	0.4	7.6
33	64	0	7.2	0.8	0.5	7.4
34	64	0	8	1	0.7	7.6
35	64	0	6.6	0.3	0.2	7.7
36	64	0	7.2	0.6	0.4	6.6
37	64	1	7	0.8	0.6	7.9
38	64	1	6.4	0.4	0.3	7.3

39	64	1	6.9	0.8	0.5	6.9
40	64	1	6.3	1.1	-	6.5
41	64	1	8.4	0.9	0.6	7.6
42	65	0	8.8	0.4	0.3	7.7
43	65	0	7.9	0.4	0.3	7.5
44	65	0	6.6	0.9	0.7	7
45	65	0	6.5	0.5	0.3	7.1
46	65	1	6.7	0.5	0.4	7.3
47	65	1	8.7	0.3	0.2	7.1
48	66	0	7.4	0.4	0.3	7.5
49	66	0	7.5	0.4	0.3	7.3
50	66	0	6.3	0.5	0.3	7.6
51	66	0	7.3	1.1	0.7	7.5
52	66	0	7.2	0.6	0.4	7.7
53	66	0	6	0.8	0.6	6.8
54	66	0	6.3	0.2	0.1	7.6
55	66	0	7.5	0.3	0.2	7.2
56	66	0	6.3	0.5	0.3	6.9
57	66	0	7	0.6	0.4	6.6
58	66	1	6	0.3	0.2	6.8
59	66	1	7.5	1	0.7	8.1
60	66	1	6.9	0.5	-	8
61	66	1	7.7	0.5	0.4	6.7
62	66	1	7.2	2.8	-	6.7
63	66	1	6.6	0.6	0.5	7.3
64	66	1	7.6	0.3	0.2	7.7
65	67	0	6.9	0.5	0.3	7.4
66	67	0	6	0.9	0.6	7.1
67	67	0	8.3	0.8	0.5	6.5
68	67	0	8.9	0.7	0.4	8
69	67	1	7.7	0.8	0.6	7.7
70	67	1	7.8	0.4	0.3	7.2
71	67	1	6.7	0.3	0.2	7.7
72	67	1	8.3	0.9	0.6	7.2
73	68	0	8.4	0.4	0.3	7.4
74	68	0	7.2	1	0.7	6.6
75	68	0	6.7	0.7	0.5	7.1
76	68	0	8.6	0.6	0.4	6.9
77	68	0	7.3	1.3	0.9	7.2

78	68	0	8	0.5	0.4	7.1
79	68	0	7.9	0.8	0.5	6.6
80	68	0	6.8	0.4	0.2	6.9
81	68	1	7	0.4	0.3	6.4
82	68	1	6.5	0.5	0.4	7.2
83	68	1	6.5	0.6	0.4	7.3
84	68	1	5.9	0.4	0.3	6.9
85	68	1	8.2	0.8	0.5	7.5
86	68	1	5.8	0.7	0.5	7.1
87	69	0	7.3	0.5	0.4	6
88	69	0	7.7	0.6	0.5	7.8
89	69	0	7	1	0.7	7.1
90	69	0	7.6	0.5	0.3	7.1
91	69	0	7.4	0.9	0.6	7.5
92	69	0	4.7	0.9	0.6	7
93	69	0	7	0.7	0.4	6.7
94	69	1	7	0.5	-	6.8
95	69	1	7.6	0.5	0.4	8.2
96	69	1	7.9	0.4	-	6.8
97	69	1	7.3	0.7	0.5	7.3
98	69	1	7.5	0.6	0.5	6.5
99	70	0	6.9	0.8	0.6	6.7
100	70	0	7.8	0.6	0.4	7.4
101	70	0	7.1	0.8	0.5	7.1
102	70	0	6.6	0.9	0.6	7.1
103	70	1	6.8	0.5	0.4	7.3
104	70	1	7	0.5	0.4	7.2
105	70	1	11	0.4	0.3	6.7
106	71	0	6.3	1.5	1.1	7.1
107	71	0	8	0.3	0.2	7.2
108	71	0	6.4	0.4	0.3	7
109	71	0	6.9	0.4	0.3	7.2
110	71	0	8.2	0.8	0.5	8.3
111	71	0	6.2	1.1	-	7.2
112	71	0	7.1	1	0.7	7.5
113	71	1	6.3	0.5	0.3	7.5
114	71	1	7.2	0.2	0.1	7.3
115	71	1	7.1	0.5	0.4	8.4
116	71	1	6.9	0.5	0.4	6.8

117	71	1	7	0.9	0.6	7.4
118	71	1	6.5	0.7	0.5	7.2
119	71	1	8	0.7	0.5	6.8
120	72	0	7.8	0.5	0.3	7.4
121	72	0	8.5	0.6	0.4	6.7
122	72	0	7.5	0.5	0.3	7
123	72	0	6.6	0.5	0.3	7.3
124	72	0	6	0.4	0.3	7.2
125	72	0	6.8	0.6	0.4	7.5
126	72	0	7.5	0.6	0.4	7
127	72	0	6.4	1.5	1.1	7.9
128	72	0	6.9	0.6	0.4	7.4
129	72	0	7.5	0.7	0.5	7.4
130	72	0	7.3	0.4	0.3	7.9
131	72	1	6.4	0.4	0.3	6.8
132	72	1	7.7	0.4	0.3	7.4
133	72	1	6.9	0.4	0.3	6.7
134	72	1	6	0.4	0.3	7.3
135	72	1	7.8	0.7	0.5	7.6
136	72	1	7.8	0.2	0.1	7.7
137	72	1	6.4	0.7	0.5	6.9
138	73	0	8.4	0.6	0.4	7.5
139	73	0	6.8	1	0.7	6.7
140	73	0	6.4	0.6	0.4	6.3
141	73	0	7.2	0.6	0.4	7.8
142	73	1	5.6	0.7	0.5	6.6
143	73	1	6.7	0.3	0.2	8.3
144	74	0	6.6	0.6	0.4	6.6
145	74	0	6.8	0.7	0.5	7.6
146	74	0	6.5	0.8	0.5	7.7
147	74	0	6.3	1.2	0.8	6.8
148	74	0	7	0.5	0.3	7.5
149	74	0	6.8	0.4	0.3	6.3
150	74	0	6.2	0.7	0.5	6.7
151	74	1	6.7	0.2	0.1	6.9
152	74	1	7.1	0.7	0.5	6.6
153	74	1	6.6	0.4	0.3	7.2
154	74	1	6.8	0.3	0.3	6.9
155	74	1	6.6	0.6	0.4	7.3

156	74	1	7.5	0.7	0.5	7.3
157	75	0	7.6	1	0.6	7
158	75	0	7.7	0.6	0.4	7.5
159	75	0	6.4	0.6	0.4	6.9
160	75	0	6.4	0.6	0.4	6.6
161	75	0	7.6	0.5	0.3	7.9
162	75	0	5.9	0.5	0.3	7.5
163	75	1	5.7	0.5	0.4	7.4
164	75	1	7.3	0.4	0.3	6.8
165	75	1	6.7	0.3	0.2	6.7
166	76	0	6.3	0.5	-	7.3
167	76	0	6.3	0.7	0.5	6.8
168	76	0	7.6	0.5	0.4	7.6
169	76	0	6.7	0.6	0.4	7.1
170	76	0	6.6	0.5	0.3	7
171	76	0	6.6	0.6	0.4	6.8
172	76	1	7	0.9	0.7	7.6
173	76	1	6.9	0.5	-	6.4
174	76	1	6.7	0.5	0.3	7.3
175	76	1	7.8	0.4	0.2	7.8
176	76	1	7.5	0.7	0.5	8.1
177	76	1	8	0.4	-	7.3
178	77	0	7.5	0.5	0.3	7.5
179	77	0	7.2	0.4	0.3	6.9
180	77	0	6.5	0.8	0.5	7.8
181	77	0	5.6	0.7	0.5	6.5
182	77	0	7.1	0.8	0.6	6.2
183	77	0	6.1	0.5	0.4	7.1
184	77	0	6.7	0.6	0.4	6.9
185	77	0	5.8	1	0.5	7.2
186	77	0	6.4	0.6	0.3	7.2
187	77	0	7.2	0.4	0.3	6.9
188	77	0	6.5	0.9	0.6	7.7
189	77	1	7.2	0.3	0.2	6.7
190	78	0	6.5	0.9	0.7	7.3
191	78	0	8.7	0.5	0.3	7.4
192	78	0	6.8	0.5	0.4	7.3
193	78	0	6.4	1.8	1.3	6.9
194	78	1	6.9	0.3	0.2	7.4

195	78	1	7.5	0.4	0.3	7.5
196	78	1	7.4	0.7	0.5	7.2
197	78	1	9.6	0.6	0.4	7.1
198	79	0	6.7	0.4	0.3	8.1
199	79	0	7.4	1.1	0.7	7.6
200	79	0	7.6	0.7	0.6	6.9
201	79	0	6.6	0.9	0.6	6.6
202	79	1	7.8	0.5 -		7.3
203	79	1	6.6	0.4	0.3	7.6
204	80	0	7.3	0.3 -		6.6
205	80	0	7.1	0.5	0.2	7.1
206	80	0	7.6	0.5	0.3	7.1
207	80	0	6.4	0.7	0.4	7.2
208	80	0	6.7	0.5	0.3	7.2
209	80	0	8.1	0.4	0.3	7.3
210	80	0	7.1	0.6	0.4	7.4
211	80	1	6.7	1.2	0.9	7.3
212	80	1	9.8	0.3 -		6
213	80	1	7.5	0.2	0.1	7.5
214	80	1	8.8	0.6	0.4	7
215	81	0	8.3	0.4	0.2	6.8
216	81	1	8.4	0.7	0.5	7.4
217	81	1	8.7	0.4	0.3	7.1
218	81	1	7.3	0.5	0.3	6.8
219	81	1	6.2	0.6	0.5	7.1
220	82	0	6.3	0.9 -		6.8
221	82	0	6	0.4 -		7.2
222	82	0	7.5	0.6	0.4	7
223	82	0	6.8	0.5	0.3	7.9
224	82	0	12.6	0.7	0.4	7.6
225	82	1	9.7	0.5	0.3	7.3
226	82	1	7.2	0.3	0.2	7.5
227	82	1	8.2	0.5 -		7.2
228	82	1	9.1	0.4	0.3	7.5
229	83	0	7.4	0.6	0.4	7.1
230	83	0	6	0.7	0.4	7.5
231	83	0	9	0.5	0.3	6.8
232	83	1	7.7	0.8 -		7.2
233	83	1	6.1	0.5	0.3	6.5

234	83	1	8.5	0.5	0.3	7.3
235	83	1	11	0.7	0.5	7.4
236	83	1	6.2	0.3	0.2	7.1
237	83	1	6.5	0.8	0.5	7.1
238	83	1	6.9	0.7	0.5	6.7
239	84	1	6.5	0.6	0.4	6.3
240	84	1	6.4	0.6	0.4	7.1
241	84	1	5.9	0.7	0.4	6.3
242	84	1	8.2	0.7	0.5	7.1
243	85	0	6.8	0.7	-	6.8
244	85	0	8.3	0.4	0.2	6.7
245	85	0	8	0.4	0.3	7.1
246	85	0	7.4	0.8	0.6	7.5
247	85	0	6.4	0.6	0.3	5.1
248	85	0	8.6	0.5	0.3	7.3
249	85	0	7.1	0.5	0.3	7.1
250	86	0	8.1	0.6	0.3	6.5
251	86	0	5.7	0.4	0.2	7.4
252	86	0	8.5	1	-	7.7
253	86	0	5.7	0.3	0.2	6.6
254	86	1	7.1	0.5	0.3	6.7
255	87	0	6.7	0.4	0.3	7.8
256	87	1	9.7	0.3	0.2	7.6
257	87	1	6.1	0.3	0.2	6.5
258	88	1	6.9	0.7	0.5	7.2
259	88	1	8.3	0.4	0.3	7.8
260	88	1	7	0.5	0.4	8
261	89	1	8	0.7	0.6	6.8
262	90	1	7.3	0.6	-	6.6
263	90	1	6.8	0.5	0.4	7.4
264	90	1	6.6	0.6	0.4	7.1
265	91	0	7.7	0.7	-	7.4
266	91	1	7	0.4	0.3	6.1
267	91	1	7.7	0.4	0.3	7.2
268	91	1	7.1	0.6	0.4	7
269	92	1	6	0.5	0.3	7.3
270	93	0	7.2	0.7	0.4	8.4
271	93	1	6	0.3	0.2	6.6
272	95	0	7.2	0.4	0.3	6.8

Albumin	Height	Weight	BMI	Soft lean mass	Skeletal muscle mass
4.3	168.8	64	22.46131	44.6	25.6
4.6	170	74.4	25.74394	50.2	29.4
4.6	165.6	73.4	26.7655	51.6	30.6
4.6	167.8	65	23.08498	50.5	29.8
3.9	153.2	52.6	22.41136	33.3	18.8
4.1	159.8	46.1	18.05292	36.2	20.4
4.3	169	84.5	29.5858	52.4	30.5
4.5	151.2	52	22.74572	31.9	17.8
4.8	154.2	62.9	26.45342	41.1	23.9
3.8	175.8	85.7	27.72957	57.7	33.3
4.3	167.8	63	22.37467	49.4	28.9
4.7	168.8	67.3	23.61947	48.4	28.3
3.4	178.2	84.3	26.5468	55.8	31.9
4.7	170.4	60.4	20.80165	43.3	25.2
4.9	168	65.5	23.2072	45.9	26.9
4.7	165.3	49.8	18.22568	35.4	19.8
5	156.4	51.9	21.21748	36.5	20.9
4.3	148	43.8	19.99635	28.7	15.6
4.8	156.6	53.3	21.7342	34.8	19.4
4.4	150.3	112.7	49.88913	48.9	28.2
4.6	153.1	49	20.90478	34.1	18.9
4.1	165.5	75	27.38201	50.5	29.7
4.6	162	66.8	25.45344	47.4	27.8
4.2	166.5	77.1	27.8116	54	32
4.7	169.7	74.5	25.86977	47.8	27.6
4.5	170.8	84.5	28.9655	52.8	30.9
4.5	163.3	49.1	18.41235	39.4	22.8
4.6	159.1	59	23.30836	36.1	20.1
4.4	147.6	77.8	35.7114	40.3	23.1
4.1	165.3	49.6	18.15248	39.3	22
4.3	155.4	64.5	26.709	36.8	20.8
4.6	167.6	60.6	21.5737	35.5	19.3
4.9	152.5	61.2	26.31551	41.9	24.5
4.6	156.5	61.1	24.94667	43.1	25.2
4.7	171.7	93.4	31.68154	61	36.4
4	158.5	60.2	23.96282	46.2	27
4.6	147.6	59.4	27.26552	35.2	20.4
4.6	149.7	50.5	22.53449	31	17.3

	4.5	156.3	66.9	27.38471	40.1	23
-		149.4	59.4	26.61247	33.1	18.4
	4.5	147.5	64	29.41683	35.2	19.8
	4.8	169.2	74.6	26.05782	48	28.1
	4.4	170.5	68.7	23.63241	50.1	29.2
	4.2	169.9	63.4	21.96355	45.6	26.4
	4.5	177.9	78.9	24.93016	59.8	35.5
	4.7	140.1	38.8	19.76767	26.4	14.5
	4.6	152	55.3	23.93525	34.7	19.8
	4.3	164.9	75.9	27.91261	46.8	26.8
	4.3	168.3	66.5	23.47758	45.3	26.3
	4.6	173.6	91.6	30.39457	55.5	33
	4.6	171.5	73.4	24.95559	51.7	30.2
	4.6	159.9	54	21.12014	36	20.3
	4.4	160.1	60.7	23.68133	45.7	26.7
	4.3	166	58.2	21.12063	44.1	25.5
	3.9	160.3	76.1	29.6154	48.2	27.8
	4.1	161.8	62.7	23.95028	38.9	21.7
	4.1	158.1	56.2	22.48395	40.5	23.5
	4	151.3	74	32.32614	38.9	22
	4.5	146.8	53.5	24.82571	30.4	16.9
-		149.6	42.7	19.0794	28.4	15.5
	4.4	162.5	62.3	23.5929	41.5	24.2
	4.4	147.7	47.5	21.77372	30.1	16.8
	4.5	152.2	64.1	27.67125	35.5	20.2
	4.7	153.8	62.3	26.33755	36	20.4
	4.4	164.8	56	20.61929	43.5	25.2
	4.6	171.2	68	23.20072	47.1	27.3
	4.3	149	42.4	19.09824	34.2	19.3
	4.8	162.7	78.5	29.65477	48.8	28.8
	4.5	154.8	50	20.86547	33.2	18.6
	4.4	156.4	65	26.57296	36.1	20.1
	4.6	152.7	59.4	25.47466	32.7	18.3
	4.6	151.1	50	21.89985	31.3	17.3
	4.5	161.1	54.9	21.15345	38.9	22.4
	4.1	167	66.7	23.91624	43.3	25
	4.3	175.6	82.8	26.85229	46.8	26.8
	4.1	166.4	54.5	19.68294	43.8	25.6
	4.6	171.4	80.8	27.50361	53.8	31.7

	4.4	171.7	78.6	26.66134	53.5	31.1
	4.2	159	59.3	23.45635	43.8	25.5
	4.5	157.6	62.3	25.08278	47.9	27.8
	4.1	153.6	48.3	20.47221	32.3	18
	4.3	162.6	89.8	33.96528	42.5	24.1
	4.4	154.1	63.8	26.86677	35.4	20
	4.4	154.6	51.2	21.42155	35.6	20.2
	4.6	158.9	54.8	21.70365	32.5	18
	4.1	161.4	62.5	23.99236	36.3	20.6
	4.1	171.6	67.6	22.95684	50	28.7
	4.7	158.7	43.7	17.35112	36	20.3
	4.3	157.5	66.2	26.68682	41.6	24
	4.6	166.4	66	23.83621	50.5	30.1
	4.7	177.1	73.6	23.46609	50.8	29.1
	3.9	164.3	67.3	24.93101	47.3	26.5
	4.1	180	83.1	25.64815	59.5	34.5
-		142.9	37.2	18.21707	29.7	15.9
	4.8	159.3	73.6	29.00322	38.9	22.1
	4.1	150.2	36.4	16.13472	28.2	15.4
	4.4	152.7	60.4	25.90352	35.2	19.7
	4.1	160.1	62.1	24.22752	36	20.2
	3.6	151.7	53.9	23.42166	35.1	20
	4.3	157.3	71.6	28.93714	43.3	24.4
	4.3	163.9	67.2	25.01563	44.8	25.9
	4.2	154.6	55.9	23.38799	43.1	24.8
	4.5	154.7	51.9	21.68636	35.4	19.6
	4.4	155.1	48.7	20.24442	34.8	19.7
	4	157.6	63.5	25.56591	37	20.6
	4.3	168	55	19.48696	40.9	23.4
	4.3	160.1	60.3	23.52527	39.8	22.9
	4.1	164.8	76.8	28.27788	51.5	30.2
	4.4	168.7	64.3	22.59336	51.2	29.7
	4.9	163.7	70.2	26.19629	44.1	25.5
-		166.8	52.5	18.8698	41.2	23.7
	4.4	168.1	66	23.35654	44.7	25.9
	4.5	151.7	59.1	25.68126	35.7	20.1
	4.5	150.4	64.9	28.69122	35.6	19.9
	4.6	154.4	53.6	22.48383	33.3	18.6
	4.2	163.2	59.7	22.41476	36	20.3

4.2	149.6	61.7	27.56906	31.9	17.7
4.4	154.4	53.1	22.27409	33.2	18.6
4.1	150	51.9	23.06667	29.1	15.9
4.6	151.2	50.1	21.91463	33.3	18.8
4.4	161.6	53.5	20.48666	46.5	26.6
4.3	166.8	72.6	26.09423	48.1	27.9
4.4	144	53.8	25.94522	27.7	15
4.5	155.4	57.3	23.72753	41.8	24
4.7	180.3	62.1	19.10294	46.2	26.5
4.5	169	80.6	28.2203	49.7	28.8
5	168.5	57.9	20.39289	46.1	26.9
4.5	162.5	54.4	20.60118	40	22.8
4.6	161.8	83	31.70451	47.7	27.8
4	169.5	78.4	27.28831	49.5	28.9
4.1	152.7	55.3	23.71631	32.1	17.9
4.3	148.1	56.9	25.94192	27.7	15
4.3	153.7	60.1	25.44056	34.1	19
4.4	146.4	55.3	25.80138	32.9	18.5
4.7	150.5	64.5	28.47651	37.7	21.4
4.6	149.3	83.9	37.63937	38.9	21.8
4.3	147.1	41.5	19.17886	30.2	16.7
4.4	169	68.9	24.12381	42.7	24.2
4.2	169.8	76.7	26.60235	47.4	27.5
3.6	160.7	51.9	20.0972	37	20.5
4.4	164.2	46.8	17.35799	35.9	20.5
4.5	160.9	49	18.9271	33.6	18.7
4.4	150	66.6	29.6	33.6	18.8
3.9	166	73.1	26.5278	46.2	26.6
4.6	156.8	46.2	18.791	34.9	19.7
4.7	155.8	70.2	28.92026	42.8	24.8
4.1	158.9	66.4	26.29785	44.4	25.1
4.4	164.1	62.4	23.17221	44.7	25.5
3.8	167	51.1	18.32264	45.5	26.6
4.1	169.7	73.4	25.4878	48.8	28.2
4.1	148	72	32.87071	32.7	17.9
4.4	155.6	47.9	19.7841	33.6	18.8
4.5	147.8	49.7	22.75137	29.8	16.4
3.7	147.3	51.6	23.78177	33.3	18.4
4.6	152.4	44.4	19.1167	33.8	19

	4.3	149.5	36	16.1072	26.9	14.5
	4.4	157	47.2	19.14885	36.1	20.2
	4.4	160.9	74.2	28.66103	42.4	24.5
	4.7	167.3	69.6	24.86665	42.7	24.1
	4.2	168	66.4	23.52608	47.6	27.1
	4.6	166	50.9	18.47148	37.3	21
	4.5	162.1	74.2	28.23826	41.1	23.1
	4.5	148.4	43.1	19.57084	29.2	16
	4.1	153.2	60.2	25.6495	34.3	19.1
	4	143.3	54.9	26.73499	29	15.9
-		164	49.9	18.55294	37.8	21.4
	4.2	167.8	75.1	26.67203	46.1	26.5
	4.2	162.5	56.5	21.39645	38.4	21.6
	4.5	171.8	80.3	27.20628	54.5	31.9
	4.3	162.2	67	25.46673	43.4	24.6
	4.3	161.2	57.8	22.24323	38.1	21.8
	4.5	147.4	46.7	21.49423	29.4	16.2
-		150.3	43.5	19.25623	28.2	15.2
	4.3	146.6	54.9	25.54491	32.2	17.7
	4.2	146.7	69.3	32.20127	32.1	17.7
	4.8	148.4	54.4	24.70194	31.2	17
-		153.4	50.8	21.58803	32.8	18.1
	4.1	174	81.7	26.98507	49.8	28.5
	4.1	160.2	56.2	21.89834	39	22.1
	4.3	163.4	60.8	22.77191	44.4	24.8
	4.2	164.4	76.2	28.19365	45.9	26.4
	3.8	168	63.8	22.60488	51	29.3
	4.2	156.2	60.6	24.83765	40.8	23.3
	4.4	159.5	58.2	22.87713	42.9	24.6
	4.4	166.4	66.5	24.01679	43.7	24.4
	4.2	158.2	67.3	26.8907	41.1	23.6
	4.3	158.8	53.7	21.29479	38.8	21.9
	4.6	162	75.9	28.9209	46.9	27.3
	4.2	149	44.9	20.22431	31.7	17.7
	4.2	165.6	65	23.70242	44.8	26
	4.1	155.4	59.2	24.51431	39.5	22.7
	4	161.1	45.9	17.68567	36.4	20.6
	4.4	169.8	60.6	21.01828	48.1	27.9
	4.9	144.8	36	17.16981	25.9	14

	3.5	144	60.6	29.22454	29	15.5
	4.4	154.4	46.7	19.58945	32.7	17.8
	3.9	146.2	60.4	28.25805	33.9	18.9
	4.1	164	63.1	23.46074	39.9	22.5
	4.5	166.9	72.1	25.88348	43.4	24.2
	4.1	157.1	57.4	23.25731	40.5	23
	4.2	170.2	62.1	21.43742	43.8	25
-		154.2	54.3	22.83658	30.6	16.8
	4.7	143.5	41.6	20.20177	26.5	14.1
-		158.3	75.5	30.12903	45.7	25.9
	3.7	166.7	60.1	21.62735	39.5	22.2
	4.1	157.8	47.6	19.11582	36.9	20.8
	4.3	166.4	69.4	25.06414	47.3	27.2
	4.5	160.2	71.2	27.7431	40.6	22.8
	4.3	158.5	59.4	23.64438	42.3	24.4
	4.4	158.8	65	25.77581	43.3	24.9
	4.4	142.3	47.4	23.40823	28.4	15.3
-		143	60.7	29.6836	28.2	15.1
	4.4	152.7	47.2	20.24249	30.8	16.9
	3.7	151.5	56.4	24.57275	31	16.9
	4.2	166.3	59.4	21.4784	35.6	19.6
	4.1	154.2	40.7	17.11692	30.1	16.6
	4.4	142.2	51.7	25.56768	29.3	16.1
	3.6	154.8	55.4	23.11894	32.8	17.9
	4.3	153.7	49.4	20.91121	31.4	17.2
-		165.9	64.6	23.47144	43.7	25.2
-		169.5	68.8	23.94689	41.2	23
	3.8	153.2	57.6	24.54172	38.1	21.2
	4.1	164	72.2	26.84414	45.4	26.1
	4.3	162.4	60.6	22.97738	34.6	19.2
	3.9	150	47	20.88889	31.2	16.9
	4.3	144.7	46.3	22.11281	27.9	15.1
-		148.1	76.3	34.7868	35.6	19.8
	4.4	147.6	44.5	20.42619	25.3	13.4
	3.8	164.8	70.4	25.92139	42.2	24
	4.4	169.7	69.8	24.23772	49.3	28.5
	3.3	162.2	66	25.08662	42	23.4
-		151.4	49.4	21.55139	29.4	16
	4.3	148.7	51.1	23.10995	30.1	16.1

4.2	150.1	51.8	22.99156	27.5	14.8
3.9	153.4	67.8	28.81237	35.7	19.9
3.8	154.8	69.5	29.003	37.6	20.7
4.3	136.5	39.4	21.14613	24.1	12.8
4.4	145.7	60.5	28.49944	32.8	18.1
4	148.4	42.5	19.29839	28.3	15.2
4.3	153	45	19.22338	33.4	18.6
3.3	150	57.8	25.68889	40.5	21.2
4.3	142	40.2	19.93652	25.2	13.4
-	165.8	68.1	24.77297	40.3	22.6
4.1	153	45.2	19.30881	32.3	17.5
4.1	154.5	56.8	23.79531	34.2	19
4.1	155.9	66	27.15512	36.4	20.4
3	165.7	57.9	21.08791	39.8	21.9
4.2	162	56	21.33821	34	18.9
3.7	158.5	57.8	23.00749	38.9	21.8
4.1	156.7	49.2	20.03674	32.2	17.8
3.8	165.5	65.1	23.76758	42.2	23.3
-	166.7	57.2	20.58377	39.3	22.1
3.5	159.6	57.8	22.69144	39.4	21.6
4.1	143	55.4	27.09179	26.8	14.2
4.4	172.4	62.4	20.99472	42.8	24.3
4.2	142.8	40.5	19.86089	30.7	16.1
3.4	145	47.1	22.4019	25.8	13.3
4.5	150.1	60.3	26.7643	34.4	19
4.2	147	66.6	30.82049	31.2	16.9
4.4	139.9	62	31.67789	27.9	14.9
4.1	147.4	50.2	23.10515	30.6	16.6
-	148	53.4	24.37911	32.7	18.1
3.9	146.2	46.8	21.89531	25.9	13.5
3.8	135	42.7	23.42936	20.8	10.5
-	159	65.5	25.90879	38.2	21.6
3.8	141.9	41	20.36194	27.2	14.3
4.4	152.6	53.2	22.8456	31.2	17.1
4.3	134.3	53.8	29.82842	26.7	14
4.2	137.3	46.6	24.71979	27.4	14.6
4.4	157.5	60.3	24.30839	41	22.6
4	147.4	41	18.87074	25.6	13.3
4.3	145.4	55.5	26.25211	28.6	15.3

Fat free mass	SMI	Body fat percentage	Fat	Renal failure	Albuminuria
47.2	6.84719	26.3	16.8	0	1
52.9	8.079585	28.9	21.5	0	1
54.4	8.532871	25.8	19	0	0
53.5	7.895062	17.7	11.5	0	0
35.3	5.986304	32.9	17.3	0	0
38.4	6.069853	16.8	7.7	0	1
55	8.57463	34.9	29.5	0	0
33.8	5.795785	34.9	18.2	0	1
43.4	7.464997	31	19.5	0	0
60.9	9.092192	29	24.8	0	1
52.2	8.253767	17.2	10.8	0	0
51.1	7.76319	24.1	16.2	0	1
59.1	8.870977	29.9	25.2	0	1
45.7	6.825971	24.3	14.7	0	0
48.5	7.266865	25.9	17	0	0
37.7	5.595795	24.3	12.1	0	0
38.7	6.218072	25.5	13.2	0	1
30.6	4.97626	30.2	13.2	0	0
36.7	6.189966	31.2	16.6	0	0
51.1	9.313819	54.7	61.6	0	1
36.1	6.262903	26.3	12.9	0	0
53.3	8.470167	28.9	21.7	0	1
50.1	8.020881	25	16.7	0	0
56.9	8.909811	26.2	20.2	0	0
50.3	7.67412	32.4	24.2	0	1
55.8	8.422275	34	28.7	0	1
41.6	6.449946	15.3	7.5	0	1
38.3	6.198443	35	20.7	1	1
42.5	7.881295	45.3	35.3	0	1
41.8	6.466821	15.8	7.8	0	0
39.1	6.704167	39.5	25.4	1	1
37.8	5.713826	37.5	22.8	0	0
44.2	7.894652	27.8	17	0	1
45.7	7.6514	25.2	15.4	0	0
64.4	9.833489	31	29	0	0
48.7	8.136214	19.2	11.5	0	0
37.2	6.738347	37.3	22.2	0	0
32.9	5.796496	34.9	17.6	0	-

42.4	7.142948	36.6	24.5	0	0
35.2	5.940764	40.7	24.2	0	0
37.1	7.409365	42	26.9	0	1
50.8	7.841798	31.9	23.8	0	0
52.9	7.83963	23	15.8	0	1
48.1	7.181457	24.1	15.3	0	0
63.1	8.840886	20	15.8	0	-
28.1	5.115139	27.4	10.7	0	0
36.7	6.587604	33.6	18.6	0	0
49.5	8.006028	34.8	26.4	0	1
47.9	7.30806	28	18.6	0	1
58.6	8.341916	36	33	0	1
54.6	8.316263	25.6	18.8	0	1
38	6.375154	29.5	16	0	0
48.1	7.943193	20.7	12.6	0	-
46.4	7.294237	20.3	11.8	0	0
50.8	8.658905	33.2	25.3	0	0
41.2	6.757263	34.3	21.5	0	-
42.9	6.941219	23.6	13.3	0	0
41	7.692748	44.5	33	1	1
32.4	5.51734	39.4	21.1	0	0
30.1	5.120607	29.6	12.6	0	1
44.1	6.91503	29.2	18.2	0	0
31.8	5.702422	33	15.7	0	0
37.5	6.717076	41.5	26.6	0	0
38	6.649914	38.9	24.3	0	0
45.9	7.036331	18.1	10.1	0	0
49.7	7.454936	27	18.3	0	0
36.1	6.328544	14.8	6.3	0	0
51.4	8.416666	34.5	27.1	0	0
35.3	5.792253	29.4	14.7	0	0
38.3	6.455184	41.1	26.7	0	1
34.7	5.802561	41.5	24.7	0	1
33.2	5.64578	33.5	16.8	0	-
41.2	6.384567	24.9	13.7	0	1
45.8	6.941805	31.4	20.9	0	1
49.4	7.384379	40.4	33.4	0	0
46.5	7.060576	14.6	8	0	0
56.8	8.479146	29.8	24	0	0

56.6	8.368133	28	22	0	0
46.3	7.689569	22	13	0	0
50.6	8.414607	18.7	11.7	0	1
34.2	5.781386	29.3	14.1	0	0
44.9	7.70838	49.9	44.9	0	0
37.5	6.459815	41.3	26.3	1 -	
37.7	6.321869	26.4	13.5	0	0
34.5	5.627899	37.1	20.3	0	0
38.4	6.510567	38.5	24.1	0	0
52.8	7.651148	21.9	14.8	0 -	
38	6.24164	13.1	5.7	0	1
43.8	7.764172	33.9	22.4	1	1
53.3	8.147652	19.3	12.7	0	0
53.7	7.709375	27	19.9	0	1
50	8.353557	25.7	17.3	1	1
63.1	8.694444	24.1	20	0	0
31.8	5.754047	14.6	5.4	0	1
41.1	6.718817	44.2	32.5	0	0
30.1	4.609921	17.4	6.3	0	1
37.2	6.488747	38.3	23.2	0	0
38.2	6.109548	38.5	23.9	0 -	
37	6.648448	31.3	16.9	0	0
45.9	8.224452	35.9	25.7	0	1
47.3	7.582861	29.6	19.9	0	1
45.2	8.192071	19.2	10.7	0	0
37.8	5.987776	27.2	14.1	0	1
36.8	6.152309	24.4	11.9	0	1
39.2	6.538432	38.2	24.3	0	0
43.2	6.409439	21.5	11.8	0	1
42.1	6.909329	30.1	18.2	0	1
54.3	8.475999	29.3	22.5	0	1
53.9	8.222156	16.1	10.4	0	0
46.6	7.302868	33.7	23.6	0	1
43.6	6.929708	17	8.9	0	0
47.2	7.396238	28.5	18.8	0	0
37.6	6.56154	36.4	21.5	0	1
37.6	6.998182	42.1	27.3	0	0
35.3	5.922978	34.2	18.3	0	1
38.3	5.894668	35.9	21.4	0	0

33.7	6.255541	45.4	28	0	0
35.2	5.826499	33.6	17.9	0	0
31.1	4.991111	40	20.8	0	1
35.2	5.927011	29.7	14.9	0	1
49.2	7.865344	8.1	4.3	0	0
50.7	7.936097	30.2	21.9	0	1
29.3	5.627894	45.6	24.5	1	1
44.1	7.888473	23	13.2	1	1
48.9	6.921354	21.2	13.2	0	0
52.5	8.122965	34.8	28.1	0	0
48.9	7.163927	15.5	9	0	1
42.3	6.627219	22.2	12.1	0	1
50.3	8.380686	39.5	32.7	0	0
52.4	8.019422	33.1	26	0	0
34	5.9012	38.5	21.3	0	0
29.5	5.010575	48.2	27.4	0	1
36.1	6.154839	40	24	0	0
34.6	6.970572	37.4	20.7	0	0
39.7	6.966811	38.4	24.8	0	0
40.9	7.877799	51.2	43	0	0
32.1	5.568801	22.6	9.4	0 -	
45	7.153111	34.7	23.9	0	0
50	7.692831	34.9	26.7	0	0
39.3	5.99431	24.3	12.6	0	1
38.1	5.85276	18.6	8.7	0	0
35.7	5.519759	27.2	13.3	1	0
35.6	6.235556	46.5	31	0	1
48.9	7.613587	33.1	24.2	0	0
37	5.926079	20	9.2	0	0
45.1	7.934533	35.7	25.1	0	0
46.9	7.885395	29.4	19.5	0	0
47.2	7.245029	24.4	15.2	0	1
47.8	7.773674	6.4	3.3	0	1
51.6	7.729679	29.7	21.8	0	0
34.5	6.656318	52.1	37.5	0	0
35.7	5.960012	25.4	12.2	0	0
31.6	5.552799	36.4	18.1	0	1
35.3	6.507725	31.6	16.3	0	0
35.8	6.217235	19.4	8.6	0	0

28.5	4.518965	21	7.5	0	0
38.4	5.955617	18.6	8.8	0	1
44.8	7.300451	39.6	29.4	0	1
45.4	6.870485	34.7	24.2	0	0
50.3	7.702664	24.2	16.1	0	0
39.4	6.154739	22.6	11.5	0	0
43.3	7.341185	41.6	30.9	1	1
31	5.171969	28.1	12.1	0	0
36.4	6.152472	39.5	23.8	0	0
30.8	5.546657	44	24.1	0	1
40	6.153331	19.9	9.9	0	0
48.7	7.898614	35.1	26.4	0	0
40.6	6.388639	28.2	15.9	0	1
57.5	8.653157	28.4	22.8	0	0
45.8	7.833869	31.7	21.2	0	1
40.3	6.434372	30.2	17.5	0	0
31.3	5.421885	32.9	15.4	0	1
30.1	5.055314	30.9	13.4	0	0
34	6.239659	38.1	20.9	0	1
34	6.077816	50.9	35.3	0	0
33.2	5.834926	38.9	21.2	0 -	
35	5.881463	31.1	15.8	0	0
52.5	7.956797	35.7	29.2	1	1
41.1	6.585089	26.9	15.1	0	0
47.3	8.288526	22.1	13.5	1	1
48.5	7.836503	36.3	27.7	0	1
54.2	8.655754	15.1	9.6	0	0
42.9	7.426704	29.3	17.7	0	0
45.6	7.330903	21.7	12.6	0	1
46.1	8.118759	30.6	20.4	0	1
43.6	7.699611	35.3	23.7	0	0
41.2	6.543091	23.3	12.5	0	0
49.6	8.139003	34.6	26.3	0	1
33.7	5.774515	24.9	11.2	0	0
47.3	7.53737	27.3	17.7	0	0
41.7	7.147247	29.5	17.5	0	1
38.5	6.033936	16.1	7.4	0	0
51.1	7.814223	15.7	9.5	0 -	
27.7	4.445072	23.2	8.3	0	0

30.7	5.574846	49.4	29.9	0	1
34.8	5.465751	25.4	11.9	0	1
35.8	6.760411	40.8	24.6	0	1
42.2	6.900654	33.1	20.9	0	1
46.1	7.413228	36.1	26	0	1
42.6	7.528238	25.7	14.8	0	0
46.3	6.955942	25.5	15.8	0	0
32.5	5.429471	40.1	21.8	0	0
28.4	4.686229	31.8	13.2	0	0
47.9	8.611714	36.6	27.6	1	1
41.7	6.427029	30.6	18.4	0	1
39	6.574075	18.1	8.6	0	0
50	7.692597	27.9	19.4	0	0
42.6	7.263081	40.2	28.6	0	1
44.6	7.272438	24.9	14.8	0	1
45.5	7.704985	29.9	19.5	0	1
30.2	5.575504	36.3	17.2	0	1
29.8	5.775344	51	30.9	0	1
32.8	5.279344	30.5	14.4	0	0
33	5.302312	41.5	23.4	0	0
37.8	5.749269	36.3	21.6	0	0
32.1	4.849094	21.2	8.6	0	0
31	5.647639	40.1	29.3	0	1
34.8	5.817292	37.2	20.6	0	1
33.4	5.354794	32.3	16	0 -	
46.1	7.150433	28.6	18.5	0	0
43.6	7.107491	36.6	25.2	1	0
40	7.383819	30.5	17.6	0	1
47.9	7.711184	33.6	24.3	0	0
36.6	6.024928	39.6	24	0	1
33.4	5.346667	29	13.6	0	1
29.8	5.105528	35.6	16.5	0	1
37.6	6.656451	50.7	38.7	0	1
27.1	4.071467	39.2	17.4	0	1
44.6	7.098925	36.7	25.8	0	1
52.1	7.851215	25.3	17.7	0	1
44.4	7.373947	32.7	21.6	0	0
31.3	5.082462	36.7	18.1	0	1
32.1	5.282274	37.1	19	0	1

29.3	4.975586	43.4	22.5	0	0
37.7	6.68039	44.3	30.1	0	1
39.9	7.060874	42.6	29.6	0	0
25.6	4.771297	35.1	13.8	1	0
34.8	6.557226	42.4	25.7	0	-
30.3	4.976715	28.6	12.2	0	1
35.4	5.984878	21.4	9.6	0	0
42.9	9.151111	25.9	14.9	0	1
27	4.255108	32.7	13.2	0	1
42.7	6.711619	37.4	25.4	0	1
34.5	5.66876	23.6	10.7	0	0
36.1	6.225322	36.4	20.7	0	0
38.5	6.422597	41.7	27.5	0	1
42.3	6.289952	27	15.6	0	1
36.1	5.620332	35.5	19.9	0	0
41.1	6.818657	28.8	16.7	0	0
34.2	5.457161	30.5	15	0	1
44.6	7.221548	31.4	20.5	1	1
41.6	6.452219	27.3	15.6	0	1
41.7	7.160759	27.9	16.1	1	0
28.5	4.763069	48.6	26.9	0	1
45.2	6.685338	27.6	17.2	0	1
32.4	6.860587	20	8.1	0	1
27.6	4.775268	41.4	19.5	0	1
36.5	6.484684	39.4	23.8	0	1
33.1	6.316812	50.2	33.5	1	1
29.5	5.431226	52.3	32.5	0	1
32.5	5.684235	35.3	17.7	0	1
34.7	5.998904	35.1	18.7	0	1
27.7	4.299528	40.7	19.1	0	1
22.2	3.978052	48	20.5	0	-
40.4	6.609707	38.3	25.1	0	0
29.3	5.05572	28.5	11.7	0	0
33.1	5.427977	37.8	20.1	0	1
28.3	5.455607	47.4	25.5	1	1
29.2	5.66009	37.4	17.4	1	1
43.2	7.687579	28.4	17.1	0	1
27.6	3.976663	32.6	13.4	0	1
30.2	5.590989	45.6	25.3	0	0

CAD	CVD	Hypertension	Hyperlipidemia	DPP4i	SGLT2i	SU
1	0	0	1	1	1	0
0	0	1	1	0	0	0
0	0	1	1	0	0	0
0	0	0	0	0	0	0
0	0	0	1	0	0	0
0	0	0	0	1	0	0
0	0	1	1	1	0	0
0	0	1	1	1	0	0
0	0	1	1	1	0	0
0	0	1	1	1	1	0
0	0	1	1	0	1	0
0	1	0	1	1	0	0
1	1	1	1	0	1	1
0	0	0	1	1	0	0
0	0	0	1	1	0	0
0	0	0	1	0	0	0
0	0	1	1	1	0	0
0	0	0	0	0	0	0
1	1	1	1	1	0	0
0	0	1	1	1	0	0
0	0	1	1	0	1	0
0	0	1	0	1	0	1
0	0	0	0	1	0	0
0	0	0	1	0	1	1
0	1	1	1	0	1	1
0	0	1	1	1	1	0
0	0	0	1	1	0	0
0	0	1	1	1	1	1
0	1	1	1	0	1	1
0	0	0	0	1	0	0
0	0	1	1	0	1	1
1	0	1	1	1	0	0
0	0	1	1	1	0	0
0	0	0	1	1	1	0
0	0	1	1	0	0	0
0	0	0	1	1	1	0
0	0	1	1	1	0	0
0	0	0	1	0	0	0

0	0	1	1	1	1	0
0	0	0	1	1	0	0
0	0	1	1	0	0	0
0	0	1	1	1	0	0
0	0	1	1	0	0	0
0	0	1	1	0	1	0
0	0	0	0	1	0	0
0	0	0	1	1	0	0
1	0	0	1	0	0	0
0	0	0	1	1	0	1
0	0	1	1	1	1	0
1	0	1	1	0	1	0
0	0	1	1	1	0	0
1	0	1	1	1	0	0
0	0	1	1	1	0	0
0	0	0	0	1	0	0
0	0	0	0	1	0	0
0	0	1	1	0	0	0
0	0	1	1	1	0	1
0	0	1	1	0	0	0
0	0	1	0	1	0	1
0	0	1	0	0	0	0
0	0	0	1	0	1	0
0	0	1	1	0	1	0
0	0	1	1	0	0	0
0	0	1	1	0	1	0
0	0	1	0	1	0	1
0	0	0	1	0	0	0
1	0	1	1	0	1	0
1	0	1	1	0	1	0
0	0	1	1	1	1	1
1	0	1	1	0	1	0
0	0	1	1	0	0	0
0	0	0	1	0	0	0
0	1	1	1	1	1	0
0	1	1	0	0	0	0
0	0	1	1	1	0	0
0	0	0	1	0	0	0
0	0	1	1	1	0	0

0	0	1	1	1	1	1
0	0	1	1	1	0	0
0	0	1	0	1	0	0
0	0	0	1	1	0	0
0	0	1	1	0	0	0
0	0	1	0	1	0	0
0	0	0	1	0	0	0
0	0	1	1	1	1	1
0	0	0	0	1	1	0
0	0	0	0	1	0	1
0	0	1	1	1	0	0
0	0	1	1	0	1	0
0	0	1	1	1	0	1
1	0	1	1	1	0	0
0	0	1	0	1	0	0
0	0	1	0	0	0	1
0	0	1	1	0	0	0
0	0	1	1	1	0	0
0	0	0	0	1	0	0
0	0	1	1	1	0	0
0	0	0	1	1	0	0
0	0	1	1	1	0	0
0	0	1	1	1	0	0
0	0	1	1	1	0	1
0	0	0	0	1	0	0
0	1	1	0	1	0	0
0	0	0	1	1	0	0
0	0	1	1	0	0	0
0	0	1	1	0	0	0
0	0	1	1	0	1	0
0	0	1	1	1	0	0
0	0	1	1	1	0	0
0	0	1	1	1	0	0
0	0	0	1	1	0	0
0	0	0	1	1	1	0
0	0	1	1	0	0	0
0	0	0	1	1	0	0
0	0	0	1	1	0	0
0	0	1	1	0	0	0

0	0	1	0	1	0	0
0	0	0	1	0	0	0
0	1	1	1	1	0	0
0	0	0	1	1	1	1
0	0	0	0	0	0	0
1	0	1	1	1	0	0
1	0	1	1	1	0	0
0	0	1	0	1	0	1
0	0	0	0	0	0	1
0	0	1	1	1	1	0
0	0	0	1	1	0	0
0	0	1	1	1	0	0
0	0	1	1	1	1	0
0	0	1	0	1	1	0
0	0	1	1	1	0	1
0	0	0	1	1	0	1
0	0	0	1	1	0	0
0	0	1	1	0	0	0
0	1	1	1	1	0	0
0	0	1	1	0	1	0
0	0	0	0	0	0	0
0	0	0	0	1	0	1
0	0	1	1	1	0	0
0	0	1	1	1	0	1
0	0	1	0	1	0	0
0	0	1	1	1	0	1
0	0	1	1	1	0	1
0	0	1	0	1	0	0
0	0	0	1	1	0	1
0	0	1	1	0	0	0
0	0	1	0	1	0	0
0	0	1	1	0	0	0
0	0	0	0	1	0	0
0	0	1	1	0	0	0
0	0	1	1	0	0	0
0	0	1	0	1	0	0
1	1	0	1	1	0	0
0	0	1	1	1	0	0
0	0	0	0	0	0	0

0	0	0	0	1	0	0
0	0	1	0	0	0	0
1	0	1	1	1	0	1
1	0	1	1	1	0	1
0	0	1	1	0	0	0
1	0	0	1	1	0	1
0	0	0	0	1	0	0
0	0	1	1	1	0	1
0	0	0	1	0	0	0
0	0	0	1	1	0	0
1	0	0	0	1	0	0
0	0	1	1	0	0	0
1	0	1	1	1	0	1
0	0	1	0	1	0	0
0	0	1	1	1	0	1
0	0	0	1	1	0	0
0	0	0	1	1	0	1
0	0	0	1	1	0	1
0	0	1	1	0	0	0
0	0	1	1	0	0	0
0	0	1	1	1	0	0
1	0	1	1	1	0	0
1	0	1	1	1	1	0
1	0	1	1	0	1	0
0	0	1	0	1	0	0
0	0	1	1	0	0	0
0	0	1	1	0	0	0
0	0	1	1	1	0	0
0	0	1	1	0	0	1
0	1	1	1	1	0	0
0	0	1	1	1	0	0
1	0	1	1	1	0	0
0	0	1	0	0	1	0
0	0	0	1	1	0	0
0	0	1	1	1	0	1
0	0	1	0	1	0	0
0	0	0	0	0	0	0
0	0	0	1	1	0	0
0	0	1	1	0	0	0

1	0	1	1	0	1	0
0	0	0	1	1	0	1
1	0	1	1	1	0	1
1	1	1	1	1	0	0
1	0	1	1	1	0	0
0	0	1	0	1	0	1
0	0	1	1	0	1	0
0	0	1	1	0	0	0
0	0	1	1	1	0	0
0	0	1	1	0	0	0
0	1	1	1	1	0	1
0	0	0	0	1	0	0
0	0	1	1	0	0	0
0	0	1	1	0	0	0
0	0	1	1	1	0	1
1	1	1	1	0	0	0
0	0	1	1	1	0	1
0	0	1	1	0	0	0
1	0	1	1	1	0	1
0	0	1	1	0	0	0
0	0	1	1	0	0	1
0	0	1	0	1	0	0
0	0	1	1	1	0	1
0	0	1	1	0	1	0
0	0	1	0	1	1	0
0	0	0	0	1	0	1
0	0	1	0	1	0	0
1	0	1	1	0	0	0
0	0	0	1	1	0	0
1	0	1	1	1	0	1
0	0	1	1	0	1	0
0	0	0	1	1	0	0
1	0	0	1	0	0	0
0	0	0	1	0	0	0
0	0	1	1	0	0	0
0	0	1	0	1	0	0
0	0	1	1	1	0	0
0	0	0	1	1	0	1
0	1	1	1	1	0	1

0	0	1	1	0	0	0
0	0	1	1	1	0	1
0	0	0	0	0	0	1
1	0	1	1	1	0	0
0	0	1	1	1	0	1
0	0	1	0	0	0	0
0	0	1	1	1	0	0
1	1	1	1	1	0	0
0	0	1	1	0	0	1
1	1	0	0	0	0	0
0	0	1	1	0	0	0
1	0	1	1	1	0	1
0	1	1	1	1	0	0
0	0	1	1	1	0	0
1	0	1	1	0	0	0
0	0	1	1	0	0	1
1	0	1	1	1	1	1
0	1	1	0	1	0	0
1	0	1	1	1	1	1
0	1	1	1	0	0	0
0	0	1	1	0	0	0
0	0	1	1	1	0	0
0	1	1	0	1	0	0
0	0	1	1	0	0	0
1	0	1	1	1	0	1
0	0	1	1	0	0	0
0	0	1	1	0	0	0
0	0	1	1	0	0	0
0	0	1	0	0	0	0
0	0	1	1	1	0	1
0	0	0	0	0	0	1
0	0	1	1	0	0	1
1	0	1	1	0	0	0
1	1	1	1	1	0	1
1	0	1	1	1	0	0
0	0	1	1	1	0	0
0	0	1	0	1	0	0
1	0	1	1	0	0	0
0	0	1	1	0	0	0

Metformin	GLP1-RA	Insulin
0	0	0
0	0	0
0	0	0
0	0	0
0	1	1
0	0	0
0	0	0
1	0	0
0	0	0
1	0	0
1	1	0
0	0	0
1	1	0
0	0	0
0	0	0
1	0	0
0	0	0
1	0	0
0	0	0
0	0	0
0	0	0
0	0	0
0	0	1
0	0	0
0	1	0
1	0	1
1	0	0
0	0	0
1	1	0
0	0	0
0	1	0
0	0	0
1	0	0
0	0	0
1	0	0
1	0	0
1	0	0
0	0	0

1	0	0
1	0	0
0	0	0
1	0	0
1	1	1
1	0	0
0	0	0
1	0	0
1	1	0
1	0	0
0	0	0
0	0	0
1	0	0
1	0	0
0	0	0
1	0	0
1	0	0
0	0	0
0	0	0
0	0	0
1	0	0
0	0	1
1	0	1
0	0	0
0	0	0
1	1	0
0	0	0
0	0	0
0	1	1
1	1	1
0	0	0
1	1	0
0	0	0
1	1	0
0	0	1
1	1	1
1	0	0
1	1	0
0	0	0

1	0	0
0	0	0
1	0	0
0	0	0
0	0	0
0	0	0
0	0	0
1	0	0
0	0	0
1	0	1
0	0	0
0	0	0
1	0	0
1	0	1
0	0	0
1	0	0
0	0	1
0	0	0
1	0	0
1	0	0
0	0	0
0	0	0
1	0	0
1	0	0
0	0	0
1	0	1
1	0	0
0	0	1
0	0	1
0	1	0
0	0	0
1	0	0
1	0	0
0	0	0
0	0	0
0	0	0
0	0	0
0	0	0
1	1	0

1	0	0
1	0	0
0	0	1
0	0	0
1	0	1
0	0	0
0	0	0
0	0	0
1	1	0
1	0	0
0	0	0
0	0	1
0	0	0
1	0	0
1	0	0
1	0	0
1	0	0
0	0	0
1	0	0
1	1	0
0	0	0
1	0	0
0	0	0
1	0	0
1	0	1
0	0	0
1	0	0
0	0	0
1	0	0
0	0	0
1	0	1
1	0	0
0	0	0
0	0	0
0	0	0
0	0	0
0	0	0
0	0	0
0	0	0

0	0	0
0	1	1
1	0	0
1	0	0
0	0	0
1	0	0
0	0	0
0	0	0
0	0	0
0	0	0
0	0	0
0	0	1
0	0	0
0	0	0
1	0	0
0	0	0
0	0	0
1	0	0
0	0	0
1	1	0
1	0	0
1	0	1
0	0	1
0	1	1
0	0	0
0	0	0
0	0	0
0	0	0
1	1	0
1	0	1
0	0	0
0	0	0
1	1	0
0	0	0
1	0	0
1	0	1
0	0	0
0	0	0
0	0	0

0	1	0
0	0	0
0	0	0
0	0	0
0	0	0
1	0	0
0	0	0
0	0	1
1	0	0
0	0	1
0	0	0
0	0	1
0	0	0
0	0	0
0	0	0
1	0	1
0	0	0
1	0	1
1	0	0
0	0	1
1	1	0
0	0	0
1	0	0
1	1	1
1	0	0
0	0	0
0	0	0
1	1	1
0	0	0
1	0	0
0	1	1
0	0	0
0	0	1
1	1	0
0	0	1
0	0	0
0	0	1
1	0	0
0	0	0

0	1	0
1	0	0
1	1	0
0	0	1
0	0	0
0	0	0
0	0	0
0	0	0
0	1	0
0	0	1
0	0	1
0	0	0
0	0	0
1	0	0
0	0	1
0	1	0
1	0	0
0	0	0
0	0	0
0	0	0
0	0	1
1	0	0
1	0	0
0	1	1
1	0	0
0	1	1
0	0	0
0	1	1
0	0	1
1	0	0
1	1	0
1	1	0
0	0	1
0	0	0
0	0	1
0	0	0
0	0	0
0	0	0
0	1	1
